# Enhancement of Copper Availability and Microbial Community Changes in Rice Rhizospheres Affected by Sulfur

**DOI:** 10.3390/molecules16021409

**Published:** 2011-02-07

**Authors:** Ji-Yan Shi, Hui-Rong Lin, Xiao-Feng Yuan, Xin-Cai Chen, Chao-Feng Shen, Ying-Xu Chen

**Affiliations:** 1 Ministry of Agriculture Key Laboratory of Non-point Source Pollution Control, Institute of Environmental Science and Technology, Zhejiang University, Hangzhou 310029, China; E-Mails: linhuirong@yahoo.com.cn (H.-R.L.); chxc8008@zju.edu.cn (X.-C.C.); ysxzt@zju.edu.cn (C.-F.S.); yingxu_chen@hotmail.com (Y.-X.C.); 2 Department of Environmental Science and Engineering, Xiamen University Tan Kah Kee College, Zhangzhou 363105, China; 3 Life Science Department, Zhejiang Chinese Medical University, Hangzhou 310053, China; E-Mail: sjyxf.ok@163.com (X.-F.Y.)

**Keywords:** sulfur transformation, PCR-DGGE, copper availability, rice rhizosphere

## Abstract

The role of sulfur on the availability of Cu and the bacterial community in rice rhizospheres was investigated by pot experiments. With sulfur addition, pH in rhizosphere soil decreased and Mg(NO_3_)_2_ extractable Cu increased significantly. The bacterial community composition also changed with sulfur addition. Some specific clones having high similarity to *Thiobacillus*, which indicated that sulfur oxidation in the rice rhizosphere could increase the availability of Cu. These results suggested that sulfur source which could provide substrate to sulfur oxidizing bacteria and enhance the availability of Cu was not a suitable sulfur fertilizer for Cu polluted soil.

## 1. Introduction

Copper (Cu) is an essential nutrient element for plants, but excessive uptake of Cu may be a great risk to plants and result in serious threats to human health through the food chain. As a result, great attention has been paid to its behavior in soil-plant system. Many studies have confirmed that the toxicity of heavy metals on plants is usually dependent on their availability [1,2,3]. It is important to study the availability of Cu in paddy soil since excessive Cu in paddy soil may be a threat to human health. Furthermore, sulfur fertilizer is widely used for the plants to increase grain yields. Paddy fields constitute a classical freshwater environment in which reduced sulfur oxidation and sulfate reduction occurs. It was confirmed that reduced sulfur was oxidized in the rice rhizosphere and thiosulfate was an important substrate for sulfur oxidizing bacteria [4,5]. Besides, sulfur has been proven to play an important role on the transformation of heavy metals [6,7,8], so it can be hypothesized that sulfur might affect the availability of Cu in paddy soil due to its relatively higher sulfur transformation rates. Hence, it is necessary to study the role of sulfur on the behavior of Cu in paddy soils.

Diversity of microbial communities is considered to be an important index of the soil quality. It is confirmed that microorganisms may play an important role on heavy metal fate in soil [9,10]. Microbes in paddy soil may be involved in sulfur transformation and then contribute to the mobility of heavy metals. Hence, it is necessary to study the role of sulfur on the microbial community composition in heavy metals contaminated soil as well as the microbial impact on the availability of heavy metals. The purpose of this study was: (1) to evaluate the role of sulfur on the availability of Cu and bacterial community composition in rice rhizosphere; (2) to investigate the effect of altered soil microbial diversity on the availability of Cu. 

## 2. Results and Discussion

### 2.1. Physicochemical property

It is believed that extractable forms of heavy metal are crucial to understand their toxicity rather than the total concentrations. Recent studies have showed that oxidation and reduction process of sulfur in soil could affect the transformations of heavy metals [7]. As a result, knowledge of how sulfur affects the availability of heavy metals in paddy soil is necessary as it is a typical sulfur cycle environment. Due to complexation with organic matter, sorption on oxides and clays, and precipitation as carbonates, hydroxides and phosphates, Cu is a relatively immobile metal in the soil [11,12,13]. In order to study the effect of sulfur on the availability of Cu, the soil pH, Mg(NO_3_)_2_ extractable Cu and plant height was tested. The results showed that sulfur addition resulted in changes of pH values and Mg(NO_3_)_2_ extractable Cu in rhizosphere soil, which indicated the important role of sulfur on the availability of Cu in paddy soil. When sulfur was added, the soil pH decreased significantly (*P* < 0.05), and Mg (NO_3_)_2_ extractable Cu also increased dramatically with sulfur treatment (*P* < 0.05) (Table 1). The reduction of soil pH has been considered to be an enhancement factor in heavy metal availability except for complexation, so the availability of Cu increased dramatically [6]. Cu resulted in high toxicity to the studied rice. As shown in Table 1, rice heights with sulfur addition were much lower than those without sulfur treatment when exposed to Cu, which further indicated the enhancement of the availability and toxicity of Cu to the rice plants.

**Table 1:** Changes of pH and Mg(NO_3_)_2_ extractable Cu concentrations in rice rhizosphere soil and rice height under Cu pollution with and without sulfur treatments. The mean of three replicates is shown with their standard deviations.

Cu added (mg kg^-1^)	pH (*P* < 0.05)		Rice height (cm)		Mg(NO_3_)_2_-Cu (mg kg^-1^) (*P* < 0.05)	
	+S	-S	+S	-S	+S	-S
0	5.05±0.17	6.01±0.06	47.67±5.51	51.00±4.00	17.08±1.02	9.20±0.07
300	5.00±0.17	5.54±0.01	47.00±2.83	50.00±4.14	23.58±2.58	12.72±0.88
1000	4.86±0.01	5.36±0.01	36.00±4.24	25.00±5.66	89.18±2.44	48.25±0.64

### 2.2. Changes in bacterial community composition measured by DGGE

**Figure 1:**
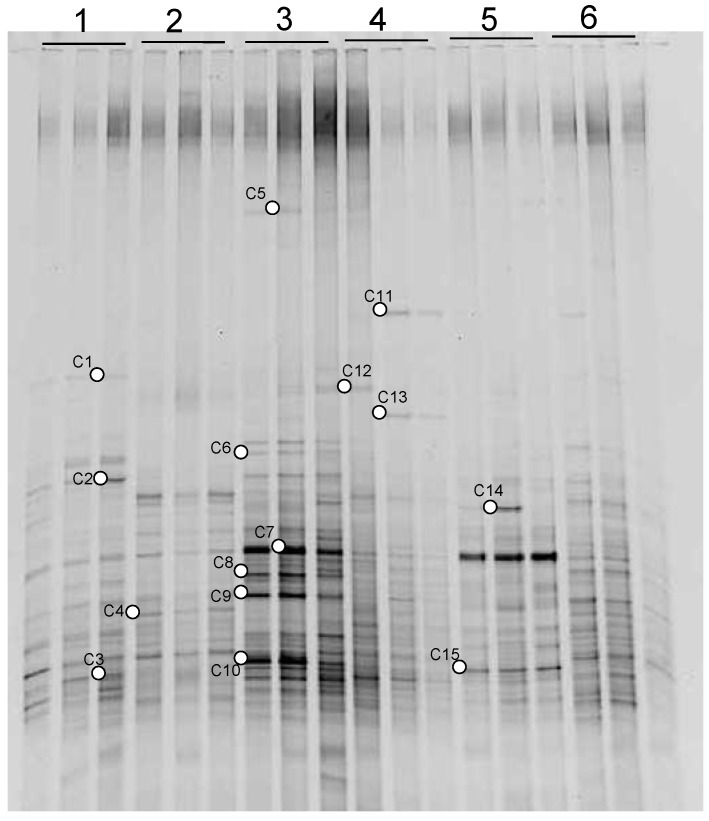
DGGE profiles of 16S rDNA fragments amplified from DNA extracted from rhizosphere soil under Cu pollution with and without sulfur treatments (1: 0 mg kg^-1^ Cu+S; 2: 0 mg kg^-1^ Cu; 3: 300 mg kg^-1^ Cu+S; 4; 300 mg kg^-1^ Cu; 5; 1000 mg kg^-1^ Cu+S; 6: 1000 mg kg^-1^ Cu).

The bacterial community composition was investigated by PCR-DGGE. As shown in Figure 1, a high reproducibility of DGGE profiles was obtained between replicates. Although lots of bands were detected in all samples, there were obvious differences among different treatments. In comparison to samples without sulfur addition, there were some new bands such as bands C7, C8, C9, C10 and C15 in those with sulfur addition (Figure 1).

**Figure 2:**
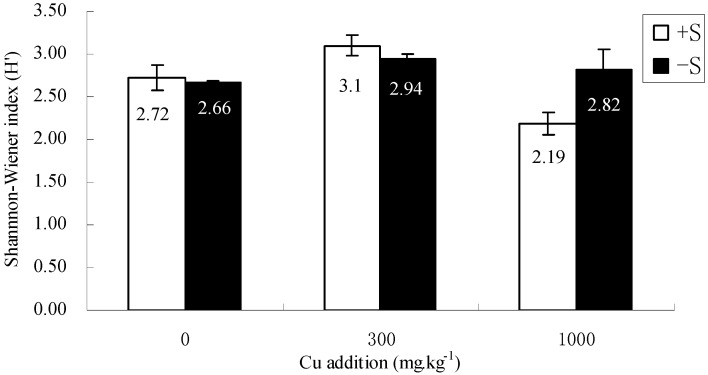
Shannon-Wiener index (*H*’) of all samples from DGGE band patterns analysis. The mean of three replicates are shown with their standard deviations.

**Figure 3:**
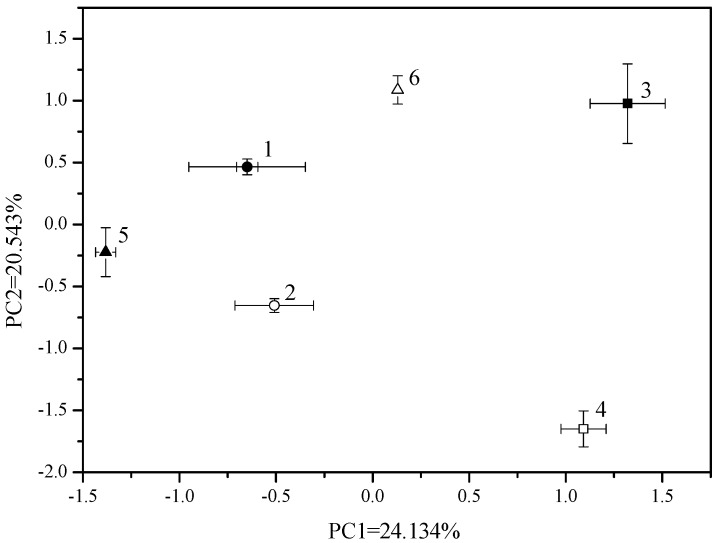
PCA analysis of DGGE patterns of 16S rRNA gene fragments amplifyied from DNA extracted form rhizosphere soil under Cu pollution with and without sulfur treatments (1: 0 mg kg^-1^ Cu+S; 2: 0 mg kg^-1^ Cu; 3: 300 mg kg^-1^ Cu+S; 4; 300 mg kg^-1^ Cu; 5; 1,000 mg kg^-1^ Cu+S; 6: 1,000 mg kg^-1^ Cu).

Subsequently, DGGE gels were interpreted using the Shannon index and principal component analysis (PCA). The Shannon index taking into account the relative intensity indicated that Shannon index increased with sulfur addition (Figure 2). PCA analysis used both band position and presence/absence as parameters. The PCA plots showed a clear separation due to different treatments which indicated an altered diversity when sulfur was added. There were significant differences (*P* < 0.05) in bacterial community composition in the rhizosphere soil between the samples with different Cu addition along the first and second principal component which explained 24.134% and 20.543% of the variation respectively. Nevertheless, it was no significant difference when the sulfur was added However, at 1,000 mg kg^-1^ Cu gradient there was also significant difference along the first principal component (Figure 3).

### 2.3. Phylogenetic analysis

The microbe-mineral interface serves as a starting point for interrogating the role of microbial organisms in geochemical transformations. Microorganisms play an important role in the biogeochemical cycle of trace elements through several microbe-mediated processes. DGGE fingerprint patterns further showed the effect of sulfur on soil microbial diversity. With sulfur addition, there were some differences in the composition of the bacterial communities in rice rhizosphere (Figure 1, Figure 2 and Figure 3), which were consistent with the study of Shi *et al.* [14], who considered that alteration in the diversity of microorganisms was a sensitive indicator of anthropogenic effects on soil ecology. Moreover, it was confirmed that microbes in soil can directly and indirectly affect heavy metal mobility [15,16]. The sulfur treatment led to changes of bacterial communities which might lead to the transformations of Cu between the soluble and insoluble phases and therefore the availability of Cu increased due to the addition of sulfur.

**Figure 4:**
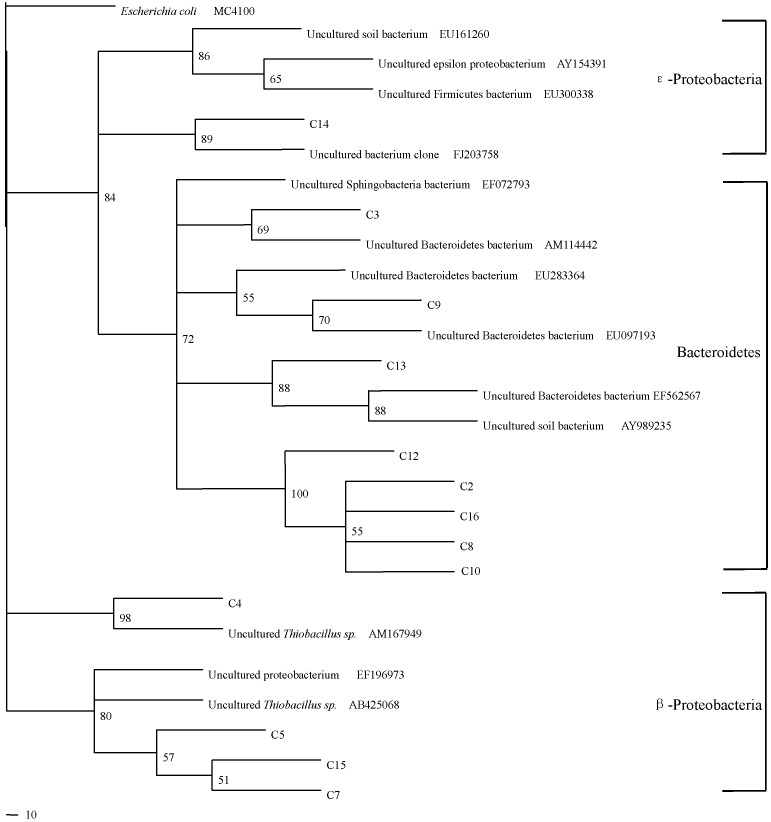
Phylogenetic tree of partial 16S rRNA gene sequences in the rhizosphere of rice under Cu pollution with and without sulfur treatments. Bootstrap support values with 1000 replicates are given along the branches.

The closest matches of the obtained sequences to the known species were determined by comparison with the RDP II database and NCBI BLAST program. Figure 4 is the phylogenetic tree of clones from the soil samples. Phylogenetic analysis of partial 16S rRNA gene sequences showed that microbial communities in the rhizosphere of tested rice were predominated by *Bacterioidetes* and *Proteobacteria*. Among these clones, C5 and C7 had 95% similarity with *Thiobacillus* AB425068 andC4 had 95% similarity to *Thiobacillus* AM167940. 

Phylogenetic analysis of partial 16S rRNA gene sequences confirmed the hypothesis above. Three specific clones had high similarity to *Thiobacillus*. This indicated that sulfur treatment provided a substrate and enhanced the activity of sulfur oxidation in the rice rhizosphere. These microorganisms in soil can use S as an energy source, then shift the abundance of soil bacterial groups. These results indicated the abundance of sulfur oxidizing bacteria in the rhizosphere soil of rice and relatively high rates of potential thiosulfate oxidation which might affect the availability of Cu. This result was consistent with previous report which concluded that about 1% of microbial flora may contribute to sulfur oxidation [4]. These microbes might be crucial for S transformation in the soil as most sulfur transformations are fundamentally controlled by biosphere processes, especially by the specialized metabolisms of microorganisms [17]. They were involved in converting reduced sulfur to sulfates, leading to the reduction of soil pH, which increased the availability of Cu in our study. Our results confirmed the important role of sulfur on soil microbial composition and their combined action on the behavior in paddy soil.

## 3. Experimental Section

### 3.1. Soil sampling and characterization

A paddy soil was collected from the top layer (0-15 cm) of a rice field in Shaoxing, Zhejiang Province, China. The soil was air-dried and sieved to a diameter of <2 mm and analyzed following the methods described by Lu [18]. Some soil properties were as follows: organic matter content, 2.26%; pH, 5.63; total Cu, 21 mg kg^-1^; total S, 0.247 g kg^-1^.

### 3.2. Experimental design and treatments

A pot experiment was carried out with 2.5 kg air dried soil mixed with 0.4 g kg^-1^ urea and 0.4 g kg^-1^ K_2_HPO_4_ as basal fertilizer for rice. Briefly, this was a factorial design with the factors Cu addition (and the levels were 0, 300, 1000 mg kg^-1^ dry soil separately) and S addition (with the level of 1 g kg^-1^ dry soils added as thiosulfate (Na_2_S_2_O_3_). Each Cu gradient has two treatments: with and without adding sulfur. All treatments were conducted in triplicate. The copper and sulfur salt was dissolved in distilled water, sprayed on the soil samples, and mixed evenly. A nylon mesh bag was placed into the middle of the pots to keep the growing roots within a relatively smaller volume and collect rhizosphere soil [19] Deionized water was added to maintain a 2-3 cm water layer overlying the surface. The treated soil was incubated for 3 months before planting rice.

### 3.3. Seedling preparation and sampling

Seeds of rice were soaked in deionized water and then germinated in soil. Seedlings were watered daily. After 40 days, three uniform seedlings were selected and transplanted to each nylon bag in plastic pot when the soil was aged after 3 months. The plants were grown in a greenhouse with average day and night temperatures of 28 and 16 °C, maintaining a 2-3 cm water layer overlying the surface with water throughout the growing season. 

After 45 days’ flood period, the plants were destructively harvested. Rice height was measured. Rhizosphere soil was collected as described by Lu [20]. Part of the soil was air dried for pH detection and Cu extraction. The rest of the soil was stored at -20 °C for DNA extraction and PCR-DGGE analysis. 

### 3.4. Cu extraction and determination

Soil availability of Cu was estimated by extracting with 0.5 M Mg(NO_3_)_2_ (1:5 w/v). The soil suspensions were shaken at 200 rpm for 2 h, centrifuged at 4,000 rpm for 10 min and then filtered. Cu concentration in soil solution was determined by FAAS (Thermo Element MK II-M6) [18]. 

Rhizosphere soil (500 mg) was extracted and purified using a bead beating method (FastDNA^TM^SPIN Kit for Soil, Bio101 Inc., USA) as described by the manufacturer. Briefly, PCR amplification was performed with primers F338GC and R518 for the V3 region of 16S rDNA [21]. PCR products were separated on a 8% arcylamide gel with a denaturing gradient of 25% to 55% denaturants for DGGE analysis at a constant voltage of 160 V (Dcode^TM ^Universal Detection System, Bio-Rad, USA). After electrophoresis, the gels were stained with SYBR^TM ^GREEN I (Sigma, USA) for 30 min and digitized with Quantity One Software (Bio-Rad, USA).

The Shannon-Wiener index was used to analyze the different treatments on the bacterial communities. It was calculated from DGGE band data as follows:

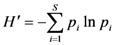
 ,

where S represents the richness or total number of bands, *p*_i _is the proportion of the total intensity accounted for by the *i*th band and ln is the natural logarithm.

### 3.5. Cloning and sequencing

Sixteen bands from the DGGE fingerprints were excised, reamplified and then purified using Qiaquick PCR Clean-up columns (Qiagen, USA). The purified products were ligated into the pMD19-T easy cloning vector (Takala, Japan) following the manufacturer’s instructions and transformed to *E. coli* DH5α competent cells. Clones grew for 12-16 hours in Luria-Bertani agar adding 100 μg mL^-1^ ampicillin, and were identified based on blue-white screening. Plamid DNA was purified with UNIQ-10 column Plasmid Mini-prep Kit (Sangon, Canada). The products were sent to the Invitrogen Corporation (USA) in China to be sequenced. Sequences were compared using the National Center for Biotechnology Information (NCBI) BLAST programs (http://www.ncbi.nlm.nih.gov/BLAST) and the Ribosomal Database Project II Chimera Check program (http://rdp.cme.msu.edu/).

### 3.6. Statistical analysis

All data were analyzed using SPSS 11.5 and Origin 7.5. Differences with and without sulfur treatment were compared statistically by Paired-Samples T Test at the 5% level with SPSS. PCA analyses of the DGGE bands were carried out based on band position and presence (presence/absence), and the correlation matrix principal component analysis and ANOVA performed by SPSS.

## 4. Conclusions

This work demonstrated the important role of sulfur on the availability of Cu as well as the microbial diversity in rice rhizosphere soil. It can be suggested from this study that reduced sulfur could provide substrate to sulfur oxidizing bacteria and enhance their activity in soil, which led to enhancement of the availability of Cu, which in turn resulted in great toxicity to rice. Since sulfur is an essential element to plants sulfur fertilizer is widely used on plants. Nowadays, Cu contamination is serious in some areas of China. Based on our study, it is suggested that reduced sulfur not be used to fertilize Cu polluted soils. 
